# (*E*)-*N*′-[1-(4-Bromo­phen­yl)ethyl­idene]benzohydrazide

**DOI:** 10.1107/S1600536809049952

**Published:** 2009-12-04

**Authors:** Hong-Yun Wang, Chuan-Gang Fan, Zhong-Nian Yang

**Affiliations:** a College of Chemistry and Chemical Technology, Binzhou University, Binzhou 256600, Shandong, People’s Republic of China

## Abstract

The asymmetric unit of the title compound, C_15_H_13_BrN_2_O, contains two independent mol­ecules with different conformations; the two aromatic rings form dihedral angles of 32.4 (4) and 27.5 (4)° in the two mol­ecules. In the crystal structure, inter­molecular N—H⋯O hydrogen bonds link mol­ecules into chains propagating in [100].

## Related literature

For the biological properties of Schiff base ligands, see: Chakraborty & Patel (1996 ▶); Jeewoth *et al.*(1999 ▶). For related crystal structures, see: Fun *et al.* (2008 ▶); Cui *et al.* (2009 ▶); Nie (2008 ▶).
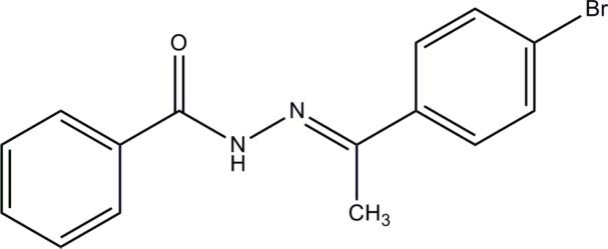


## Experimental

### 

#### Crystal data

C_15_H_13_BrN_2_O
*M* *_r_* = 317.18
Monoclinic, 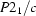

*a* = 9.9770 (11) Å
*b* = 31.487 (3) Å
*c* = 8.7613 (9) Å
β = 96.1040 (10)°
*V* = 2736.8 (5) Å^3^
*Z* = 8
Mo *K*α radiation
μ = 3.00 mm^−1^
*T* = 298 K
0.49 × 0.18 × 0.12 mm

#### Data collection

Bruker SMART APEX CCD area-detector diffractometer
Absorption correction: multi-scan (*SADABS*; Sheldrick, 1996 ▶) *T*_min_ = 0.321, *T*_max_ = 0.715
13558 measured reflections
4804 independent reflections
1961 reflections with *I* > 2σ(*I*)
*R*_int_ = 0.079

#### Refinement

*R*[*F* ^2^ > 2σ(*F* ^2^)] = 0.064
*wR*(*F* ^2^) = 0.189
*S* = 1.02
4804 reflections
345 parameters
H-atom parameters constrained
Δρ_max_ = 0.47 e Å^−3^
Δρ_min_ = −0.44 e Å^−3^

### 

Data collection: *SMART* (Siemens, 1996 ▶); cell refinement: *SAINT* (Siemens, 1996 ▶); data reduction: *SAINT*; program(s) used to solve structure: *SHELXS97* (Sheldrick, 2008 ▶); program(s) used to refine structure: *SHELXL97* (Sheldrick, 2008 ▶); molecular graphics: *SHELXTL* (Sheldrick, 2008 ▶); software used to prepare material for publication: *SHELXTL*.

## Supplementary Material

Crystal structure: contains datablocks I, global. DOI: 10.1107/S1600536809049952/cv2651sup1.cif

Structure factors: contains datablocks I. DOI: 10.1107/S1600536809049952/cv2651Isup2.hkl

Additional supplementary materials:  crystallographic information; 3D view; checkCIF report

## Figures and Tables

**Table 1:** Hydrogen-bond geometry (Å, °)

*D*—H⋯*A*	*D*—H	H⋯*A*	*D*⋯*A*	*D*—H⋯*A*
N3—H3⋯O1^i^	0.86	2.20	2.943 (8)	144
N1—H1⋯O2	0.86	2.23	3.000 (8)	149

Symmetry code: (i) 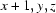.

**Fig. 1.**
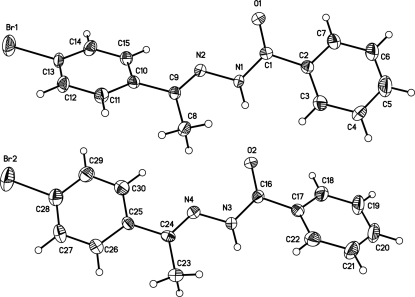
A view of (I) showing the atomic numbering scheme and 30% probability displacement ellipsoids.

## supplementary crystallographic information

### Comment

Schiff base compounds are known as having strong anticancer activity (Chakraborty & Patel, 1996) and various biological properties (Jeewoth *et al.*, 1999). Herewith we present the title compound (I) - a new Shiff base compound.

In (I) (Fig. 1), the bond lengths an angles are normal and are comparable to the values observed in similar compounds (Nie *et al.*, 2008; Fun *et al.*, 2008; Cui *et al.*, 2009). The asymmetric unit of (I), contains two independent molecules A and B, respectively. In molecule A, two aromatic rings form a dihedral angle of 32.4 (4) °, while in molecule B this angle is 27.5 (4) °. The C=N bond lengths in the molecules are 1.293 (9)° and 1.272 (9)° (C9=N2,C24=N4), showing the double-bond character.

Weak intermolecular N—H···O hydrogen bonds (Table 1) link molecules into one-dimensional chain propagated in direction [100].

### Experimental

Benzohydrazide (5.0 mmol), 20 ml e thanol and 4-bromoacetophenone (5.0 mmol) were mixed in 50 ml flash. After refluxing 3 h, the resulting mixture was cooled to room temperature, and recrystalized from ethanol, and afforded the title compound as a crystalline solid. Elemental analysis: calculated for C_15_H_13_BrN_2_O: C 56.80, H 4.13, N 8.83%; found: C 56.78, H 4.24, N 8.69%.

### Refinement

All H atoms were placed in geometrically idealized positions (N—H 0.86 Å, C—H=0.93–0.96 Å) and treated as riding on their parent atoms, with *U*_iso_(H) = 1.2–1.5 *U*_eq_(C, N).

### Crystal data

**Table:**

C_15_H_13_BrN_2_O	*F*(000) = 1280
*M**_r_* = 317.18	*D*_x_ = 1.540 Mg m^−^^3^
Monoclinic, *P*2_1_/*c*	Mo *K*α radiation, λ = 0.71073 Å
*a* = 9.9770 (11) Å	Cell parameters from 1649 reflections
*b* = 31.487 (3) Å	θ = 2.6–22.4°
*c* = 8.7613 (9) Å	µ = 3.00 mm^−^^1^
β = 96.104 (1)°	*T* = 298 K
*V* = 2736.8 (5) Å^3^	Block, colourless
*Z* = 8	0.49 × 0.18 × 0.12 mm

### Data collection

**Table:**

Bruker SMART APEX CCD area-detector diffractometer	4804 independent reflections
Radiation source: fine-focus sealed tube	1961 reflections with *I* > 2σ(*I*)
graphite	*R*_int_ = 0.079
phi and ω scans	θ_max_ = 25.0°, θ_min_ = 1.3°
Absorption correction: multi-scan (*SADABS*; Sheldrick, 1996)	*h* = −11→10
*T*_min_ = 0.321, *T*_max_ = 0.715	*k* = −37→37
13558 measured reflections	*l* = −9→10

### Refinement

**Table:**

Refinement on *F*^2^	Primary atom site location: structure-invariant direct methods
Least-squares matrix: full	Secondary atom site location: difference Fourier map
*R*[*F*^2^ > 2σ(*F*^2^)] = 0.064	Hydrogen site location: inferred from neighbouring sites
*wR*(*F*^2^) = 0.189	H-atom parameters constrained
*S* = 1.02	*w* = 1/[σ^2^(*F*_o_^2^) + (0.0544*P*)^2^ + 6.194*P*] where *P* = (*F*_o_^2^ + 2*F*_c_^2^)/3
4804 reflections	(Δ/σ)_max_ < 0.001
345 parameters	Δρ_max_ = 0.47 e Å^−^^3^
0 restraints	Δρ_min_ = −0.44 e Å^−^^3^

### Special details

**Table:**

Geometry. All e.s.d.'s (except the e.s.d. in the dihedral angle between two l.s. planes) are estimated using the full covariance matrix. The cell e.s.d.'s are taken into account individually in the estimation of e.s.d.'s in distances, angles and torsion angles; correlations between e.s.d.'s in cell parameters are only used when they are defined by crystal symmetry. An approximate (isotropic) treatment of cell e.s.d.'s is used for estimating e.s.d.'s involving l.s. planes.
Refinement. Refinement of *F*^2^ against ALL reflections. The weighted *R*-factor *wR* and goodness of fit *S* are based on *F*^2^, conventional *R*-factors *R* are based on *F*, with *F* set to zero for negative *F*^2^. The threshold expression of *F*^2^ > σ(*F*^2^) is used only for calculating *R*-factors(gt) *etc*. and is not relevant to the choice of reflections for refinement. *R*-factors based on *F*^2^ are statistically about twice as large as those based on *F*, and *R*- factors based on ALL data will be even larger.

### Fractional atomic coordinates and isotropic or equivalent isotropic displacement parameters (Å^2^)

**Table:**

	*x*	*y*	*z*	*U*_iso_*/*U*_eq_	
Br1	0.05576 (10)	0.04761 (3)	0.12825 (13)	0.0742 (4)	
Br2	0.53233 (12)	0.04532 (3)	0.13223 (14)	0.0925 (5)	
N1	0.1623 (6)	0.30472 (16)	0.1860 (7)	0.0375 (17)	
H1	0.2450	0.3123	0.2070	0.045*	
N2	0.1275 (6)	0.26193 (18)	0.1697 (8)	0.0414 (18)	
N3	0.6642 (6)	0.30098 (17)	0.1957 (7)	0.0415 (18)	
H3	0.7454	0.3084	0.2272	0.050*	
N4	0.6303 (6)	0.25836 (18)	0.1760 (7)	0.0375 (17)	
O1	−0.0567 (5)	0.32437 (15)	0.1524 (7)	0.0512 (16)	
O2	0.4486 (5)	0.32125 (15)	0.1277 (6)	0.0465 (15)	
C1	0.0627 (8)	0.3340 (2)	0.1678 (9)	0.037 (2)	
C2	0.1063 (8)	0.3795 (2)	0.1712 (9)	0.036 (2)	
C3	0.2317 (9)	0.3936 (3)	0.2308 (11)	0.056 (3)	
H3A	0.2965	0.3740	0.2693	0.067*	
C4	0.2627 (9)	0.4362 (3)	0.2342 (13)	0.077 (3)	
H4	0.3476	0.4454	0.2750	0.092*	
C5	0.1660 (12)	0.4652 (3)	0.1765 (13)	0.084 (4)	
H5	0.1857	0.4941	0.1776	0.100*	
C6	0.0444 (10)	0.4515 (3)	0.1191 (11)	0.066 (3)	
H6	−0.0198	0.4711	0.0792	0.079*	
C7	0.0123 (8)	0.4089 (2)	0.1179 (10)	0.052 (2)	
H7	−0.0741	0.4002	0.0806	0.063*	
C8	0.3318 (7)	0.2479 (2)	0.3464 (9)	0.039 (2)	
H8A	0.4062	0.2526	0.2873	0.058*	
H8B	0.3543	0.2257	0.4196	0.058*	
H8C	0.3132	0.2736	0.3996	0.058*	
C9	0.2105 (7)	0.2354 (2)	0.2421 (9)	0.032 (2)	
C10	0.1756 (7)	0.1897 (2)	0.2133 (9)	0.034 (2)	
C11	0.2275 (8)	0.1584 (2)	0.3126 (9)	0.041 (2)	
H11	0.2867	0.1656	0.3979	0.049*	
C12	0.1921 (8)	0.1161 (2)	0.2866 (10)	0.045 (2)	
H12	0.2287	0.0953	0.3537	0.053*	
C13	0.1045 (8)	0.1051 (2)	0.1637 (10)	0.036 (2)	
C14	0.0528 (8)	0.1356 (2)	0.0611 (10)	0.046 (2)	
H14	−0.0047	0.1281	−0.0253	0.055*	
C15	0.0875 (7)	0.1777 (2)	0.0884 (9)	0.036 (2)	
H15	0.0505	0.1984	0.0210	0.044*	
C16	0.5674 (8)	0.3303 (2)	0.1644 (9)	0.0331 (19)	
C17	0.6123 (7)	0.3751 (2)	0.1748 (10)	0.037 (2)	
C18	0.5576 (8)	0.4041 (2)	0.0657 (10)	0.051 (2)	
H18	0.4952	0.3948	−0.0137	0.061*	
C19	0.5939 (10)	0.4464 (3)	0.0728 (13)	0.074 (3)	
H19	0.5593	0.4653	−0.0029	0.089*	
C20	0.6835 (10)	0.4602 (3)	0.1961 (14)	0.073 (3)	
H20	0.7081	0.4887	0.2034	0.088*	
C21	0.7347 (10)	0.4324 (3)	0.3050 (13)	0.071 (3)	
H21	0.7933	0.4420	0.3874	0.085*	
C22	0.7009 (8)	0.3897 (3)	0.2950 (11)	0.056 (3)	
H22	0.7380	0.3709	0.3695	0.068*	
C23	0.8370 (8)	0.2420 (2)	0.3406 (11)	0.052 (2)	
H23A	0.9101	0.2458	0.2791	0.077*	
H23B	0.8582	0.2194	0.4125	0.077*	
H23C	0.8231	0.2678	0.3953	0.077*	
C24	0.7126 (7)	0.2313 (2)	0.2401 (9)	0.034 (2)	
C25	0.6731 (8)	0.1857 (2)	0.2140 (9)	0.036 (2)	
C26	0.7538 (8)	0.1525 (2)	0.2651 (11)	0.057 (3)	
H26	0.8374	0.1581	0.3188	0.068*	
C27	0.7146 (10)	0.1107 (3)	0.2391 (12)	0.065 (3)	
H27	0.7719	0.0886	0.2734	0.078*	
C28	0.5913 (10)	0.1022 (3)	0.1627 (11)	0.058 (3)	
C29	0.5083 (9)	0.1343 (3)	0.1057 (10)	0.057 (3)	
H29	0.4250	0.1285	0.0518	0.069*	
C30	0.5519 (8)	0.1757 (3)	0.1305 (10)	0.057 (3)	
H30	0.4973	0.1977	0.0892	0.069*	

### Atomic displacement parameters (Å^2^)

**Table:**

	*U*^11^	*U*^22^	*U*^33^	*U*^12^	*U*^13^	*U*^23^
Br1	0.0932 (8)	0.0335 (5)	0.0912 (9)	−0.0095 (5)	−0.0117 (6)	−0.0017 (5)
Br2	0.1330 (11)	0.0416 (6)	0.1011 (11)	−0.0177 (6)	0.0043 (8)	−0.0026 (6)
N1	0.032 (4)	0.021 (3)	0.058 (5)	−0.003 (3)	0.001 (3)	−0.001 (3)
N2	0.033 (4)	0.028 (3)	0.063 (6)	−0.002 (3)	0.005 (4)	0.004 (3)
N3	0.032 (4)	0.027 (3)	0.064 (6)	0.000 (3)	−0.002 (4)	0.001 (3)
N4	0.035 (4)	0.037 (4)	0.041 (5)	−0.003 (3)	0.005 (4)	0.001 (3)
O1	0.034 (3)	0.038 (3)	0.083 (5)	−0.005 (3)	0.008 (3)	−0.003 (3)
O2	0.028 (3)	0.038 (3)	0.072 (5)	−0.004 (2)	0.000 (3)	−0.004 (3)
C1	0.031 (5)	0.035 (4)	0.045 (6)	−0.001 (4)	0.006 (4)	0.000 (4)
C2	0.036 (5)	0.034 (4)	0.040 (6)	0.000 (4)	0.008 (4)	0.000 (4)
C3	0.045 (6)	0.046 (5)	0.075 (8)	0.003 (4)	0.000 (5)	−0.018 (5)
C4	0.045 (6)	0.048 (6)	0.137 (11)	−0.015 (5)	0.006 (6)	−0.030 (6)
C5	0.096 (9)	0.037 (6)	0.123 (11)	−0.003 (6)	0.036 (8)	0.001 (6)
C6	0.067 (7)	0.033 (5)	0.099 (9)	0.001 (5)	0.019 (6)	0.008 (5)
C7	0.050 (5)	0.036 (5)	0.072 (7)	0.005 (4)	0.006 (5)	0.010 (4)
C8	0.043 (5)	0.033 (4)	0.039 (6)	−0.006 (4)	−0.003 (4)	0.003 (4)
C9	0.034 (5)	0.029 (4)	0.035 (6)	0.001 (3)	0.005 (4)	0.008 (4)
C10	0.033 (5)	0.032 (4)	0.036 (6)	0.002 (3)	0.005 (4)	0.005 (4)
C11	0.045 (5)	0.039 (4)	0.037 (6)	−0.003 (4)	−0.004 (4)	−0.004 (4)
C12	0.054 (6)	0.034 (4)	0.044 (6)	−0.005 (4)	−0.004 (5)	0.014 (4)
C13	0.045 (5)	0.021 (4)	0.044 (6)	−0.002 (3)	0.008 (4)	0.002 (4)
C14	0.051 (6)	0.039 (5)	0.044 (6)	−0.011 (4)	−0.003 (4)	−0.003 (4)
C15	0.041 (5)	0.036 (4)	0.032 (6)	0.001 (4)	0.003 (4)	0.009 (4)
C16	0.035 (5)	0.032 (4)	0.033 (6)	−0.002 (4)	0.008 (4)	−0.009 (4)
C17	0.033 (5)	0.038 (4)	0.042 (6)	0.003 (4)	0.015 (4)	−0.010 (4)
C18	0.052 (6)	0.040 (5)	0.059 (7)	−0.003 (4)	−0.001 (5)	0.005 (4)
C19	0.085 (8)	0.031 (5)	0.108 (10)	−0.006 (5)	0.018 (7)	0.001 (5)
C20	0.075 (8)	0.035 (5)	0.114 (11)	−0.010 (5)	0.031 (7)	−0.024 (6)
C21	0.064 (7)	0.055 (6)	0.094 (9)	−0.016 (5)	0.012 (6)	−0.036 (6)
C22	0.043 (6)	0.058 (6)	0.068 (7)	0.002 (4)	0.004 (5)	−0.015 (5)
C23	0.035 (5)	0.048 (5)	0.070 (7)	0.000 (4)	−0.002 (5)	0.000 (5)
C24	0.024 (5)	0.043 (5)	0.036 (6)	0.005 (4)	0.015 (4)	−0.002 (4)
C25	0.035 (5)	0.033 (4)	0.042 (6)	0.007 (4)	0.010 (4)	0.007 (4)
C26	0.046 (6)	0.042 (5)	0.081 (8)	0.005 (4)	0.001 (5)	0.009 (5)
C27	0.070 (7)	0.035 (5)	0.090 (9)	0.013 (5)	0.007 (6)	0.005 (5)
C28	0.064 (7)	0.042 (5)	0.070 (8)	−0.001 (5)	0.008 (6)	−0.005 (5)
C29	0.054 (6)	0.051 (5)	0.065 (7)	−0.006 (5)	−0.003 (5)	−0.013 (5)
C30	0.050 (6)	0.049 (5)	0.071 (8)	0.003 (4)	−0.004 (5)	−0.004 (5)

### Geometric parameters (Å, °)

**Table:**

Br1—C13	1.891 (7)	C12—C13	1.358 (10)
Br2—C28	1.895 (8)	C12—H12	0.9300
N1—C1	1.353 (9)	C13—C14	1.379 (10)
N1—N2	1.395 (7)	C14—C15	1.382 (9)
N1—H1	0.8600	C14—H14	0.9300
N2—C9	1.293 (9)	C15—H15	0.9300
N3—C16	1.343 (8)	C16—C17	1.481 (9)
N3—N4	1.390 (7)	C17—C22	1.380 (11)
N3—H3	0.8600	C17—C18	1.390 (10)
N4—C24	1.272 (9)	C18—C19	1.380 (10)
O1—C1	1.223 (8)	C18—H18	0.9300
O2—C16	1.228 (8)	C19—C20	1.396 (13)
C1—C2	1.496 (9)	C19—H19	0.9300
C2—C7	1.366 (10)	C20—C21	1.356 (13)
C2—C3	1.376 (10)	C20—H20	0.9300
C3—C4	1.378 (10)	C21—C22	1.385 (11)
C3—H3A	0.9300	C21—H21	0.9300
C4—C5	1.384 (13)	C22—H22	0.9300
C4—H4	0.9300	C23—C24	1.482 (10)
C5—C6	1.334 (12)	C23—H23A	0.9600
C5—H5	0.9300	C23—H23B	0.9600
C6—C7	1.380 (10)	C23—H23C	0.9600
C6—H6	0.9300	C24—C25	1.498 (10)
C7—H7	0.9300	C25—C26	1.367 (10)
C8—C9	1.490 (10)	C25—C30	1.382 (10)
C8—H8A	0.9600	C26—C27	1.385 (11)
C8—H8B	0.9600	C26—H26	0.9300
C8—H8C	0.9600	C27—C28	1.363 (12)
C9—C10	1.496 (9)	C27—H27	0.9300
C10—C11	1.380 (9)	C28—C29	1.368 (11)
C10—C15	1.382 (10)	C29—C30	1.385 (10)
C11—C12	1.388 (9)	C29—H29	0.9300
C11—H11	0.9300	C30—H30	0.9300
			
C1—N1—N2	118.4 (6)	C15—C14—H14	120.5
C1—N1—H1	120.8	C14—C15—C10	121.8 (7)
N2—N1—H1	120.8	C14—C15—H15	119.1
C9—N2—N1	115.7 (6)	C10—C15—H15	119.1
C16—N3—N4	118.5 (6)	O2—C16—N3	123.3 (6)
C16—N3—H3	120.7	O2—C16—C17	120.9 (7)
N4—N3—H3	120.7	N3—C16—C17	115.8 (7)
C24—N4—N3	117.1 (6)	C22—C17—C18	118.6 (7)
O1—C1—N1	122.6 (7)	C22—C17—C16	122.0 (8)
O1—C1—C2	121.2 (7)	C18—C17—C16	119.3 (8)
N1—C1—C2	116.2 (7)	C19—C18—C17	121.4 (9)
C7—C2—C3	118.3 (7)	C19—C18—H18	119.3
C7—C2—C1	117.0 (7)	C17—C18—H18	119.3
C3—C2—C1	124.6 (7)	C18—C19—C20	118.6 (9)
C4—C3—C2	121.0 (8)	C18—C19—H19	120.7
C4—C3—H3A	119.5	C20—C19—H19	120.7
C2—C3—H3A	119.5	C21—C20—C19	120.4 (9)
C3—C4—C5	119.2 (9)	C21—C20—H20	119.8
C3—C4—H4	120.4	C19—C20—H20	119.8
C5—C4—H4	120.4	C20—C21—C22	120.7 (10)
C6—C5—C4	119.7 (9)	C20—C21—H21	119.6
C6—C5—H5	120.1	C22—C21—H21	119.6
C4—C5—H5	120.1	C17—C22—C21	120.3 (9)
C5—C6—C7	121.2 (9)	C17—C22—H22	119.9
C5—C6—H6	119.4	C21—C22—H22	119.9
C7—C6—H6	119.4	C24—C23—H23A	109.5
C2—C7—C6	120.5 (8)	C24—C23—H23B	109.5
C2—C7—H7	119.8	H23A—C23—H23B	109.5
C6—C7—H7	119.8	C24—C23—H23C	109.5
C9—C8—H8A	109.5	H23A—C23—H23C	109.5
C9—C8—H8B	109.5	H23B—C23—H23C	109.5
H8A—C8—H8B	109.5	N4—C24—C23	124.7 (7)
C9—C8—H8C	109.5	N4—C24—C25	115.3 (7)
H8A—C8—H8C	109.5	C23—C24—C25	119.9 (7)
H8B—C8—H8C	109.5	C26—C25—C30	116.8 (7)
N2—C9—C8	124.5 (6)	C26—C25—C24	123.2 (8)
N2—C9—C10	114.4 (7)	C30—C25—C24	120.0 (7)
C8—C9—C10	121.1 (6)	C25—C26—C27	121.9 (9)
C11—C10—C15	117.8 (7)	C25—C26—H26	119.0
C11—C10—C9	121.2 (7)	C27—C26—H26	119.0
C15—C10—C9	121.0 (7)	C28—C27—C26	119.4 (8)
C10—C11—C12	120.7 (8)	C28—C27—H27	120.3
C10—C11—H11	119.7	C26—C27—H27	120.3
C12—C11—H11	119.7	C27—C28—C29	121.0 (8)
C13—C12—C11	120.5 (7)	C27—C28—Br2	120.3 (7)
C13—C12—H12	119.8	C29—C28—Br2	118.7 (7)
C11—C12—H12	119.8	C28—C29—C30	118.2 (8)
C12—C13—C14	120.2 (7)	C28—C29—H29	120.9
C12—C13—Br1	120.5 (6)	C30—C29—H29	120.9
C14—C13—Br1	119.3 (6)	C25—C30—C29	122.7 (8)
C13—C14—C15	119.1 (8)	C25—C30—H30	118.7
C13—C14—H14	120.5	C29—C30—H30	118.7

### Hydrogen-bond geometry (Å, °)

**Table:**

*D*—H···*A*	*D*—H	H···*A*	*D*···*A*	*D*—H···*A*
N3—H3···O1^i^	0.86	2.20	2.943 (8)	144
N1—H1···O2	0.86	2.23	3.000 (8)	149

Symmetry codes: (i) *x*+1, *y*, *z*.
